# Digital comparison of healthy young adults and borderline patients engaged in non-suicidal self-injury

**DOI:** 10.1186/s12991-015-0088-5

**Published:** 2015-12-21

**Authors:** Rachel Stroehmer, Marc A. Edel, Steffi Pott, Georg Juckel, Ida S. Haussleiter

**Affiliations:** LWL-Institute of Mental Health, LWL-University Hospital Bochum, Alexandrinenstr. 1, 44791 Bochum, Germany; Dept. of Psychiatry, LWL-University Hospital Bochum, Alexandrinenstr. 1, 44791 Bochum, Germany

**Keywords:** BriefTEMPS-M, Anonymous online questionnaire, Hidden body area, Borderline personality disorder

## Abstract

**Background:**

It still remains unclear whether non-suicidal self-injury (NSSI) in young adult populations represents an actual symptom leading to psychiatric illness, constitutes a disorder itself or is rather a cultural peer influence. The purpose of this web-based qualitative cross-sectional study was to characterize NSSI (type of injury, frequency, tools, body parts, circumstances) in 50 patients with borderline personality disorder (NSSI + BPD) in direct comparison with 50 age and gender matched non-clinical young adults (NSSI − BPD), all of them currently or previously engaged in NSSI.

**Methods:**

Self-harming participants completed an open-access, anonymous 75-items questionnaire including the temperament questionnaire briefTEMPS-M.

**Results:**

The mean age of NSSI onset was 20.56 ± 6.36 (NSSI + BPD) and 17.5 ± 9.28 years (NSSI − BPD), respectively (*p* = 0.261). NSSI − BPD participants (1) rarely sought out medical treatment (*p* < 0.001) and differed significantly from BPD patients; They (2) reported more often fear and disappointment as feelings preceding their self-harm (*p* < 0.001 each); (3) cut themselves in more locations (*p* = 0.005) and (4) in rather hidden areas (lower limb, proximal) (*p* = 0.002); (5) had lower depressive temperament scores (*p* = 0.007); and (6) scored generally fewer character traits “at risk” (*p* = 0.043) with a lower total score (*p* = 0.018). NSSI tended to onset slightly earlier in life and in different shape when BPD was absent.

**Conclusions:**

Our findings support current approaches of early NSSI recognition and identification of risk profiles. Further prospective studies, which have to be sufficiently large and longitudinal, are needed and of great importance.

## Background

Non-suicidal self-injury (NSSI) is defined as “deliberate, self-inflicted destruction of body tissue without suicidal intent and for purposes not socially sanctioned” [1]. NSSI in young adult populations might be an underestimated public health issue and causes significant familial, medical and psychiatric concern [2]. It remains uncertain whether this behavior pattern represents an actual symptom pointing to psychiatric illness, constitutes a disorder in itself or has to be considered a reflection of peer culture influence [3].

The prevalence of self-injury in young adults, including the nonclinical population, may be increasing [4]. One out of three self-injurers reports an onset of self-injurious behavior in childhood, with a peak incidence in mid- to late-adolescence. In most studies, self-cutting is the most common form of self-injury, followed by burning and self-hitting or banging; common locations are the forearms, wrists, and thighs [5]. Klonsky [6] reported that many individuals who engage in NSSI practice more than one of the methods mentioned above. Moreover, lifetime frequency and variety of methods can be taken as indicators of NSSI severity [7]. So far, only few of the young adults affected are seeking medical attention [8].

Over the past few years, several quantitative studies evaluated NSSI in schools [9–11] and reported inconsistent prevalence between 3 and 37 % [12] for children and adolescents in Germany.

According to DSM-V, borderline personality disorder (BPD) is manifested by a pervasive pattern of instability of interpersonal relationships, self-image and affects, and a marked impulsivity beginning in early adulthood, and occurring in a variety of contexts. NSSI as self-mutilating behavior is considered primarily a function of high BPD symptoms and one of the diagnostic BPD criteria [13, 14]. As NSSI is often present in individuals who are not diagnosed with BPD, the former was considered a distinct category in DSM-V. The precise definition of NSSI might lead to a better comparability of study outcomes with regard to NSSI by a more standardized research [15, 16].

Variables such as emotional dysregulation [17], low social support [18, 19], global psychological distress [8], cognitive style [20], sense of meaning in life [21] and mental health history [5] seem to amplify NSSI and may also mediate the relationship between NSSI and future suicidal thoughts and behaviors [4].

Discussing and reflecting on emotional problems generally seem relieving, but whom one chooses to confide in about such mental distress matters. Talking to peers may actually exacerbate such distress for young people [22], whereas communication with parents is considered a protective factor for later suicidal behavior [4].

So far, there is little evidence on the relationship between BPD symptoms and the functions of NSSI in youth [23]. The purpose of this web-based cross-sectional study was to characterize NSSI in a clinical sample of adult BPD outpatients engaged in NSSI (NSSI + BPD) in comparison with an age- and gender-matched non-clinical sample of adults also endorsed in NSSI (NSSI − BPD). It was hypothesized that these two groups differ in self-harm characteristics (type of injury, frequency, tools, body parts, circumstances) and display different underlying temperament traits (anxious, cyclothymic, depressive, hyperthymic and irritable trait). Focusing on a non-clinical sample may help identify individuals at risk at an early stage, increase awareness, and enable better treatment.

## Methods

### Participants

We evaluated a clinical and non-clinical sample of young adults, either currently or previously engaged in NSSI. Current NSSI was defined as still on-going at the time of this study. The clinical sample (NSSI + BPD) consisted of 50 unrelated German outpatients (82 % female, mean age 26.8 ± 6.53 years) diagnosed with a BPD according to DSM-IV criteria (301.83) and currently enrolled in outpatient psychiatric treatment in the LWL-University-Hospital Bochum, Germany. The Ethics Committee of the Medical Faculty of the Ruhr-University Bochum approved the study, and written informed consent was obtained from all participants. The non-clinical sample (NSSI − BPD) was matched for age (mean age 26.84 ± 6.23), sex, and ethnicity, and recruited within a larger web-based NSSI survey (data unpublished). This survey exclusively comprised young adults who currently or previously endorsed in NSSI and were not diagnosed with BPD.

### Measures

BPD diagnosis was confirmed by the complete Structured Clinical Interview for DSM-IV [24] providing moderate to excellent inter-rater agreement for Axis I disorders, and excellent inter-rater agreement for most categorically and dimensionally measured personality disorders [25].

A questionnaire comprising 40 items was prepared to assess personal information, educational background, family structures, attachment style, and the participants’ social network. Nosological items dealt with psychiatric diagnoses, risk behavior, and substance abuse. Intention, methods, and tools of self-injury were queried in detail. All these items were scored using a five-gradation Likert-type response scale.

The second part of the questionnaire with 35 items consisted of the briefTEMPS-M [26], which is the short German version of the TEMPS-A auto-questionnaire [27]. It evaluates depressive, hyperthymic, cyclothymic, irritable and anxious temperaments and affective disorders in a Likert-type response format and with randomized items.

Examples of the questions included are „People tell me I am unable to see the lighter side of things“, „my mood often changes for no reason“, „I go back and forth between feeling overconfident and feeling unsure of myself”, „The way I see things is sometimes vivid, but at other times lifeless”.

Temperament was analyzed with a dimensional score of the respective temperament scale (percentage of agreement). The temperament trait was coded as clinically present if patients agreed to more than 70 % of the items in accordance with Ozgürdal et al. [28]. Thus, each trait was evaluated relatively (exceeding cutoff) and absolutely (total score). The internal consistency (*α*) for the briefTEMPS-M varies between 0.69 and 0.84 and the test–retest reliability is 0.49–0.72 [26].

### Procedure

Within the study period of 4 months, all BPD outpatients currently or previously engaged in NSSI (NSSI + BPD) were personally invited to participate in the study while waiting for their doctor’s appointment. They subsequently received the link to the study webpage to participate anonymously on their computers at home. Inclusion criteria were: NSSI at present or in anamnesis, being 18 years and older, suffering from BPD, and no acute suicidal ideation. For the non-clinical control group, the link to the study webpage was distributed via online social network (facebook^**®**^) and German bulletin boards. Exclusion criterion was a diagnosis of BPD and participants were asked whether they currently used the mental health system. Online social networks are an anonymous and client-centered tool to directly get in touch with young people. Nowadays, manifold platforms allow users to share information online with people all over the world such as Twitter^®^, facebook^®^ and Google+^®^. The world’s largest social network today is facebook^®^ with 1.44 billion monthly active users as of March 2015 [29].

After reading and digitally signing a statement of informed consent, participants completed the above-mentioned 75-item-questionnaire. Participation was voluntary and anonymous, and all study subjects were assured that their data would be kept confidential. If participants were not engaged in NSSI (both groups) or had a diagnosis of BPD (NSSI − BPD), the questionnaire ended automatically.

The service-platform LimeService was used to prepare, run and evaluate the web-based survey. Distributed under GNU General Public License and written in PHP, it is a free and open source online survey application based on several databases like MySQL. Results were exported and further edited in Microsoft^®^ Excel and IBM^®^ SPSS^®^ Statistics 21.0.

Continuous data are presented with means (M), the standard deviation (SD), and categorical data with number of subjects and percentage. Temperament was analyzed with a dimensional score of the respective temperament scale (percentage of agreement). For comparison of categorical and continuous variables, *Chi* square tests, *t* tests or Fisher’s exact test were used where appropriate. A *p* value of less than 0.05 was interpreted as significant.

## Results

### Study population

82 % of the participants were female, and the mean age was 26.8 ± 6.53 (NSSI + BPD) and 26.84 ± 6.23 years (NSSI − BPD), respectively (range 18–43 years; *p* = 0.975). The general qualification for university entrance differed significantly between NSSI + BPD (38 %) and NSSI − BPD (62 %) (*p* = 0.016), as well as final graduation from university with 4 (NSSI + BPD) and 22 % (NSSI − BPD) (*p* = 0.015).

### NSSI

**Fig. 1:**
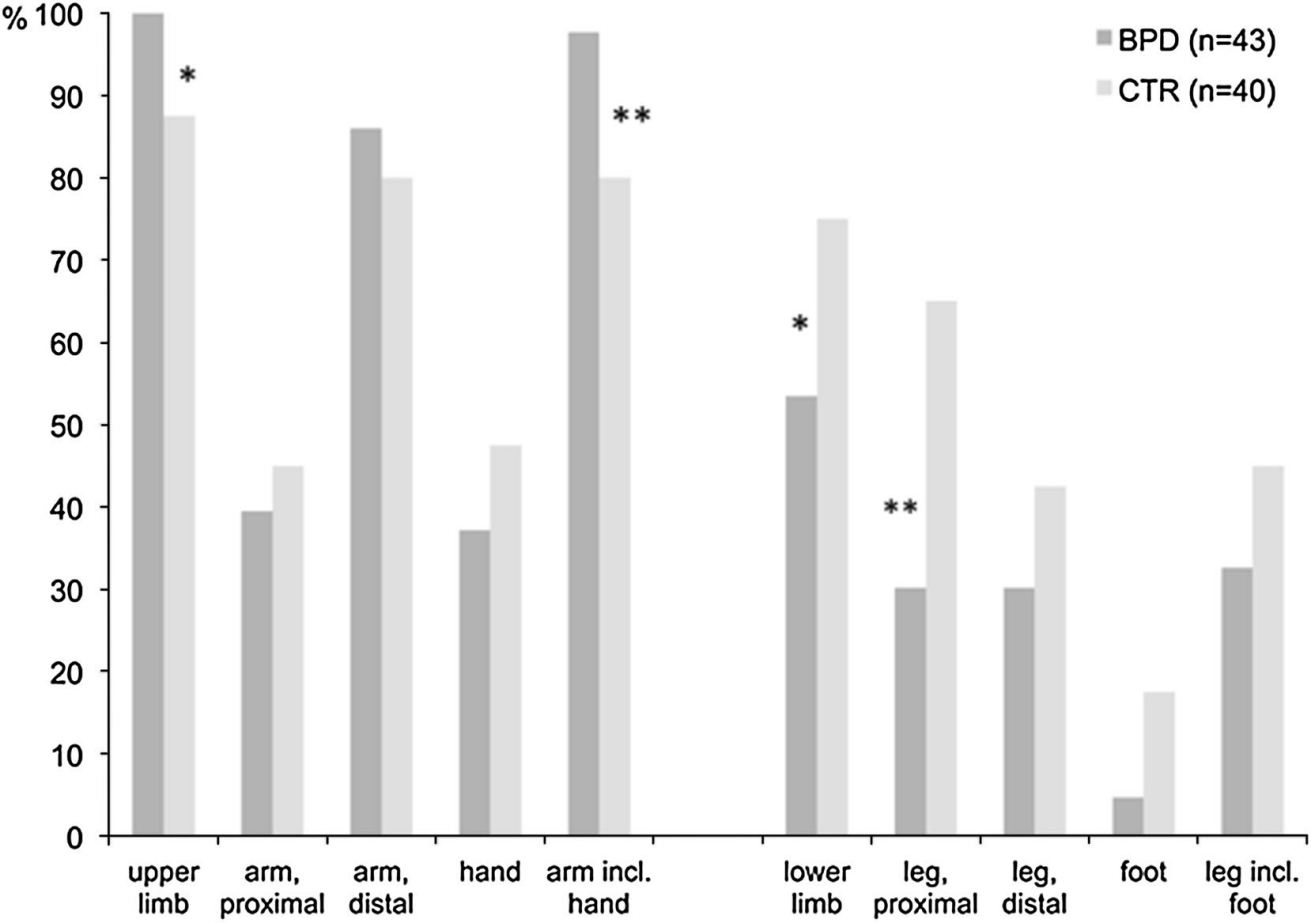
Preferred localizations of self-cutting in NSSI + BPD (*n* = 43) and NSSI − BPD (*n* = 40) subjects

In both groups, there were more than half of the subjects currently engaged in NSSI (64 NSSI + BPD, 56 % NSSI − BPD; *p* = 0.414), the remaining subjects reported to have injured themselves in the past, and meanwhile ceased this habit completely. To cease the behavior once and for all, NSSI − BPD participants declared significantly more often relationships as a reason (*p* = 0.049). Relationships in this context were defined as having a new partner, the ending of a harmful relationship, as well as birth or the growing up of own children. The mean age of NSSI onset was 20.56 ± 6.36 (NSSI + BPD, range 8–33 years) and 17.5 ± 9.28 years (NSSI − BPD, range 6–43 years), respectively (*p* = 0.261). The claim of mental health services differed significantly between groups: 38 % of the NSSI − BPD group (for other reasons than NSSI) and all NSSI + BPD patients (recruited from a psychiatric outpatient center) underwent treatment (*p* < 0.001). On a trend level, NSSI + BPD patients (40 %) talked more often with other persons than therapists about their self-injuries than NSSI − BPD subjects (22 %, *p* = 0.052). Strain, inner emptiness, aggression, and sadness recurrently occurred in both groups prior to each NSSI session. Fatigue (*p* = 0.006), disappointment, and fear (*p* < 0.001 each) occurred significantly more often in the control group (NSSI − BPD). The desire to experience oneself more intensely was the main reason to start NSSI in both groups (94 NSSI + BPD, 76 % NSSI − BPD; *p* = 0.051) and only a minority declared attention-seeking behavior (2 NSSI + BPD, 6 % NSSI − BPD; *p* = 0.548). Experiencing oneself more intensely was defined as gaining back the sense for one’s own body and replenishing one’s inner emptiness (see Table 1). NSSI + BPD subjects (1.96 ± 1.195) cut themselves in significantly fewer areas than the NSSI − BPD subjects (2.38 ± 1.861; *p* = 0.005). NSSI + BPD patients cut themselves significantly more often on the upper limbs (*p* = 0.017), whereas NSSI − BPD subjects significantly more often chose their lower limbs (*p* = 0.042) (see Fig. 1). NSSI − BPD subjects indicated to cut themselves significantly more often in places not showing when fully clothed such as proximal lower limbs (*p* = 0.002), whereas NSSI + BPD patients significantly more often chose more visible locations such as distal upper limbs including hands (*p* = 0.010).

**Table 1:** NSSI characteristics

	NSSI + BPD	NSSI − BPD	*Chi*-square^a^ */t* test^b^	*df*	*p* value
Basics					
Still lasting/current (n, %)	32 (64 %)	28 (56 %)	0.667^a^	1	0.414
Age of onset (mean ± SD)	20.56 ± 6.36	17.5 ± 9.28	−1.507^b^	58	0.137
Duration (mean ± SD)					
In months, current	66.27 ± 87.61	105.8 ± 84.55	1.774^b^	58	0.081
In months, ceased	57.09 ± 77.49	59.58 ± 43.44	0.136^b^	44	0.892
Each time in minutes	20.04 ± 25.3	16.34 ± 15.8	−0.878^b^	98	0.382
Use of mental health services (n, %)	50 (100 %)	19 (38 %)	44.928^a^	1	<0.001***
Talking about NSSI with (n, %)					
Other than therapist	17 (34 %)	10 (20 %)	2.486^a^	2	0.088
Parents	3 (6 %)	1 (2 %)	3.835^a^	2	0.147
Open presentation (n, %)	8 (16 %)	5 (10 %)	0.796^a^	1	0.554
Reason for NSSI (n, %)					
More attention	1 (2 %)	3 (6 %)	1.203^a^	2	0.548
Intense feeling	47 (94 %)	38 (76 %)	5.943^a^	2	0.051
Reason for interruption (each time) (n, %)					
Feelings changed	41 (82 %)	42 (84 %)	0.071^a^	1	0.790
Bleeding	20 (40 %)	14 (28 %)	1.604^a^	1	0.205
Exhausted	8 (16 %)	15 (30 %)	2.767^a^	1	0.096
Caught	8 (16 %)	8 (16 %)	0.000^a^	1	1.000
Reason for complete discontinuation (n, %)					
Relationship	1 (4.5 %)	7 (29.2 %)	4.901^a^	2	0.049*
Therapy	8 (36.4 %)	4 (16 %)	2.903^a^	2	0.234
Need	1 (4.5 %)	14 (56 %)	14.561^a^	2	0.001**
Feelings prior to action (n, %)					
Strain	42 (84 %)	37 (74 %)	1.507^a^	1	0.220
Inner emptiness	37 (74 %)	30 (60 %)	2.216^a^	1	0.137
Aggression	37 (74 %)	29 (58 %)	2.852^a^	1	0.091
Sadness	31 (62 %)	34 (68 %)	0.396^a^	1	0.529
Fear	3 (6 %)	25 (50 %)	24.008^a^	1	<0.001***
Fatigue	3 (6 %)	14 (28 %)	8.575^a^	1	0.006**
Pleasure	1 (2 %)	1 (2 %)	0.000^a^	1	1.000
Disappointment	1 (2 %)	22 (44 %)	24.901^a^	1	<0.001***
Methods					
Number of methods (mean ± SD)	1.78 ± 0.93	1.56 ± 0.91	−1.196^b^	98	0.235
Cutting (n, %)	43 (86 %)	40 (80 %)	0.638^a^	1	0.424
Hitting (n, %)	24 (48 %)	20 (40 %)	0.649^a^	1	0.420
Burning (n, %)	15 (30 %)	16 (32 %)	0.047^a^	1	0.829
Trichotillomania (n, %)	7 (14 %)	2 (4 %)	3.053^a^	1	0.160
Self-cutting localizations	n = 43	n = 40			
Number of localizations (mean ± SD)	1.96 ± 1.195	2.38 ± 1.861	1.343^b^	98	0.005**
Upper limb (n, %)	43 (100 %)	35 (87.5 %)	4.487^a^	2	0.017*
Arm, proximal	17 (39.5 %)	18 (45 %)	0.044^a^	2	0.834
Arm, distal	37 (86 %)	32 (80 %)	1.177^a^	2	0.555
Arm, distal incl. hand	42 (97.7 %)	32 (80 %)	5.198^a^	2	0.023*
Hand (n, %)	16 (37.2 %)	19 (47.5 %)	2.257^a^	2	0.323
Lower limb	23 (53.5 %)	30 (75 %)	8.019^a^	2	0.042*
Leg, proximal	13 (30.2 %)	26 (65 %)	7.104^a^	2	0.008**
Leg, distal	13 (30.2 %)	17 (42.5 %)	3.673^a^	2	0.159
Leg, distal incl. foot	14 (32.6 %)	18 (45 %)	0.735^a^	2	0.391
Foot	2 (4.7 %)	7 (17.5 %)	5.760^a^	2	0.056

NSSI + BPD *n* = 50; NSSI−BPD *n* = 50; *p < 0.05; **p < 0.01; ***p < 0.001

^a^ *Chi*-square; ^b^ *t* test

### Temperament (character traits) and risk behavior

Table 2 shows the results of the briefTEMPS-M and risk behavior in both groups. The mean number of traits exceeding cutoff was 1.52 ± 1.165 (NSSI + BPD) versus 1.02 ± 1.270 (NSSI − BPD; *p* = 0.043), the total score was 75.64 ± 19.01 (NSSI + BPD; range 38–119) versus 66 ± 21.17 (NSSI − BPD; range 29–113), respectively (*p* = 0.018). All but the depressive trait were equally distributed in both groups. All NSSI + BPD patients (100 %) and one-third of NSSI − BPD subjects exceeded the 70 % cutoff of depressive items (*p* = 0.041). NSSI + BPD patients had a higher total score for all five temperaments and groups differed significantly regarding the depressive trait (*p* = 0.007). Out of five possible risk behaviors, the study participants on average performed 2.36 ± 1.59 (NSSI + BPD) and 2.36 ± 1.84 (NSSI − BPD; *p* = 1.000) different ones. Regarding each high-risk behavior in detail, driving at high speed (56 %), staying in obviously dangerous places (44 %), and consuming substances with unknown effects (40 %) occurred more often in the control group, whereas NSSI + BPD patients more often drank too much alcohol (54 %), took drugs (30 %), changed sexual partners more frequently (24 %), and admitted more often to have unprotected sex (20 %).

**Table 2:** Brief TEMPS-M and risk behavior

	NSSI + BPD	NSSI − BPD	*Chi*-square^a^ */t* test^b^	*df*	*p* value
Temperament (mean ± SD)					
Number of traits exceeding cutoff	1.52 ± 1.165	1.02 ± 1.270	−2.052^b^	98	0.043*
Total score of traits	75.6 ± 19.01	66 ± 21.17	−2.396^b^	98	0.018*
Depressive trait					
Existent (n, %)	25 (50 %)	15 (30 %)	4.167^a^	1	0.041*
score (mean ± SD)	19.4 ± 6.2	15.9 ± 6.2	−2.772^b^	98	0.007**
Cyclothymic trait					
Existent (n, %)	21 (42 %)	15 (30 %)	1.563^a^	1	0.211
Score (mean ± SD)	17.8 ± 5.26	15.48 ± 7.59	−1.776^b^	98	0.079
Hyperthymic trait					
Existent (n, %)	3 (6 %)	2 (4 %)	0.211^a^	1	1.000
Score (mean ± SD)	9.8 ± 6.55	9.74 ± 4.69	−0.053^b^	98	0.958
Irritable trait					
Existent (n, %)	12 (24 %)	10 (20 %)	0.233^a^	1	0.629
Score (mean ± SD)	13.9 ± 6.33	11.18 ± 7.97	−1.889^b^	98	0.062
Anxious trait					
Existent (n, %)	15 (30 %)	9 (18 %)	1.974^a^	1	0.160
Score (mean ± SD)	14.78 ± 6.58	13.68 ± 6.21	−0.860^b^	98	0.392
Risk behavior					
Number of risk behaviors (mean ± SD)	2.36 ± 1.59	2.36 ± 1.84	0.000^b^	98	1.000
Minimum 3 risk behaviors (n, %)	22 (44 %)	19 (38 %)	0.372^a^	1	0.542
Drinking too much alcohol (n, %)	27 (54 %)	24 (48 %)	0.360^a^	1	0.548
Severity of alcohol use (mean ± SD)^c^	0.98 ± 1.22	0.98 ± 1.29	0.000^b^	98	1.000
Taking drugs (n, %)	15 (30 %)	13 (26 %)	0.198^a^	1	0.656
Drug use, severity (mean ± SD)^c^	0.52 ± 0.93	0.50 ± 0.91	−0.109^b^	98	0.914
Driving at high speed (n, %)	22 (44 %)	28 (56 %)	1.440^a^	1	0.230
Speed, severity (mean ± SD)^c^	0.94 ± 1.25	1.34 ± 1.45	1.476^b^	98	0.143
Staying in obviously dangerous places (n, %)	15 (30 %)	22 (44 %)	2.102^a^	1	0.147
Places, severity (mean ± SD)^c^	0.52 ± 0.95	0.78 ± 1.15	1.232^b^	98	0.221
Substances with unknown effects (n, %)	17 (34 %)	20 (40 %)	0.386^a^	1	0.534
Substances, severity (mean ± SD)^c^	0.66 ± 1.12	1.02 ± 1.42	1.408^b^	98	0.162
Changing sexual partners frequently (n, %)	12 (24 %)	7 (14 %)	1.624^a^	1	0.202
Having unprotected sex frequently (n, %)	10 (20 %)	4 (8 %)	2.990^a^	1	0.148

NSSI + BPD *n* = 50; NSSI − BPD *n* = 50; *p < 0.05; **p < 0.01; ***p < 0.001

^a^ *Chi*-square; ^b^ *t* test; ^c^ Severity: 0–4

## Discussion

The main findings of this cross-sectional study were that control participants (1) rarely sought out medical treatment and in comparison to NSSI + BPD patients; (2) reported more often fear and disappointment as feelings preceding their self-harm; (3) cut themselves in more locations; (4) cut themselves in rather hidden areas (lower limb, proximal); (5) had lower depressive temperament scores; and (6) scored generally fewer briefTEMPS-M character traits “at risk” with a lower total score. Owing to the survey’s anonymity based on an automatic generation of aliases, a high level of openness was possible and expected.

The mean age at the time of the interview was 26.8 years. The average age of onset of NSSI among young adolescents is 12–14 years [30], even though NSSI affects individuals from all age groups [31]. Young adults aged between 18 and 25 years are believed to be at the greatest risk for engaging in such behavior [32]. The indicated age of NSSI onset in our study was 20.56 ± 6.36 and 17.5 ± 9.28 years for NSSI + BPD and control participants (NSSI − BPD), respectively. Even though the groups did not differ significantly in age of NSSI onset, the control participants apparently started earlier with their self-harm behavior. This is all the more interesting, since they sought out professional help for other reasons than their NSSI.

In 2005, Whitlock and colleagues conducted the first large survey-based study of self-injury in a population of 3.069 students. Using a web-based survey, the team examined self-reports of self-injurious practices, age of onset, forms, severity, intention, and help-seeking behavior. One main outcome, consistent with our study, was that NSSI happened in individuals who had never been in therapy for any reason, and that only few of them disclosed their behavior and sought help [8]. The study at hand confirms the communication difficulties of self-injurers, since only 12 % of the control participants talked about their behavior whereas almost half of them (44 %) performed self-harm without confiding in anyone.

Even though NSSI can occur in the course of psychiatric disorders, recent studies suggest that the social circumstances and experiences of the person concerned are more crucial in explaining what leads to self-harm than a diagnosed psychiatric disorder [33]. NSSI might be a particular reaction to emotional distress and not necessarily herald a manifest disease. On the other hand, the assumption that patients endorsing NSSI are more attention seeking and manipulative and less in genuine need of mental health might lead to an underestimation of the severity and potential lethality of NSSI [30, 34].

People engaged in NSSI often report greater emotional dysregulation than those without NSSI history and NSSI has been associated with an emotion regulation function and trait emotion dysregulation among people who self-injure [35]. We accordingly discovered that strain, inner emptiness, aggression and fear led to participants’ self-injuries.

To our knowledge, no empirical studies investigating in detail the body locations chosen for self-injury have yet been conducted. In our study, NSSI − BPD subjects chose more different locations, which were at the same time easily hidden and concealed from the detection of others, whereas NSSI + BPD subjects deliberately cut themselves in more exposed regions.

A number of risk factors for self-injury have been identified including depressed mood, increased anxiety, low self-esteem and cognitions that focus upon self-failure [36, 37]. Depression and anxiety in adolescence are associated with an increased incidence of self-harm in young adulthood [38] but generally measured current depressive or anxiety traits lacked discriminative ability in distinguishing between history of and ongoing NSSI in our study.

NSSI + BPD subjects might suffer from axis I disorders more frequently than the NSSI − BPD controls. Turner et al. (2015) observed that BPD patients showed greater diagnostic comorbidity, particularly for anxiety disorders, but did not differ from participants without BPD in rates of mood, substance or psychotic disorders. The NSSI + BPD group in that study reported more severe depressive symptomatology, suicidal ideation and emotion dysregulation than the NSSI − BPD group [39]. An effect of such possible comorbidities on our outcome parameter (NSSI characteristics) is possible but seems less significant. There is no clinical explanation as to why an existing psychiatric comorbidity should, for example, alter the localization pattern of self-cutting. Regarding temperament traits and risky behaviors, the two groups in the current study did not differ significantly. NSSI + BPD patients generally scored higher—in terms of total briefTEMPS-M score—than control participants (NSSI − BPD) and groups differed highly significant only in depressive temperament (*p* = 0.007). Highest score possible for one trait was 28 (7 questions with 4 severity grades each). Therefore, the mean total scores can be similar, since the scores of all participants are included in the calculation. For the calculation of clinically present temperament traits on the other side, only those with a score greater or equal to 20 points (70 % cutoff) were included. Thus, the discrepancy can be explained by the fact that more NSSI + BPD patients scored 20 and higher (exceeding cutoff), whereas more NSSI − BPD subjects scored in the double-digit range (adding up to a relatively higher total score in this group).

Cyclothymic, irritable and especially depressive temperaments might represent an important marker of vulnerability to NSSI in young adults [40] and the higher rate of dominant affective temperaments found among NSSI + BPD patients might reflect the suggested relationship between affective temperaments and full-blown mood disorders [41, 42], which are often comorbid to BPD [43].

The present study has certain limitations that need to be taken into account when interpreting its results: The size of the subgroups was relatively small with 50 participants each, limiting the representativeness and reliability of the data and also precluding meaningful subgroup analysis. The advantage of an anonymous online survey allowed for more openness and a higher rate of participation when talking about such a delicate and potential embarrassing topic such as NSSI.

Manifest BPD has to be absent in NSSI − BPD group, but due to the design of the study (using an anonymous online questionnaire) a further clinical interview was not feasible. Surely, when anonymized, honesty has to be assumed. If participants indicated a BPD diagnosis, the questionnaire ended automatically. Overall, we registered 516 accesses to the webpage, whereof 328 questionnaires were complete. The complete sample of controls, out of which the group in the current study was drawn (matching the BPD patients in age and gender), comprised over 300 non-clinical young adults. Based on the total population of more than 300 young adults, it seems highly unlikely that all of our matched controls “secretly” suffered from a BPD and did not report it.

The questionnaire to qualitatively assess NSSI and accompanying factors has been developed for this pilot study and not been validated or standardized beforehand. Available standardized NSSI instruments were either not in an appropriate format for the purpose of this study (open-ended question inquiring about methods used for NSSI in the Self-Harm Behavior Questionnaire by Fliege et al. [44]) or did not exist yet in a translated and validated German version (such as the self-injurious thoughts and behaviors interview by Fischer et al. [45]).

Nevertheless, the study benefits from examining a wide range of NSSI characteristics, including method type, number of methods, location, reasons for discontinuation, age at onset, duration of engagement, reasons for engagement, feelings experienced before engagement which would not have been possible with a pre-built questionnaire.

Both samples were heterogeneous in terms of the types of self-injurious behaviors they were engaged in (e.g., substance use, abusive relationships, and risky behavior). People who engage in some of these behaviors may be different from those who engage in others.

In conclusion, our findings support current approaches of early NSSI recognition and identification of risk profiles. Further prospective studies are needed and of great importance. They have to be sufficiently large and longitudinal to directly focus on the limitations named above.

## Ethics, consent, and permissions

The Ethics Committee of the Medical Faculty of the Ruhr-University Bochum approved of the study, and written informed consent was obtained from all participants. The study has, therefore, been performed in accordance with the ethical standards laid down in the 1964 Declaration of Helsinki and its later amendments.
